# Antimicrobial resistance in aeromonads and new therapies targeting quorum sensing

**DOI:** 10.1007/s00253-024-13055-z

**Published:** 2024-02-13

**Authors:** Blake Neil, Gabrielle L. Cheney, Jason A. Rosenzweig, Jian Sha, Ashok K. Chopra

**Affiliations:** 1 Department of Microbiology and Immunology, Medical Branch, University of Texas, Galveston, TX 77555 USA; 2 John Sealy School of Medicine, Medical Branch, University of Texas, Galveston, TX 77555 USA; 3 Department of Biology, Texas Southern University, Houston, TX 77004 USA

**Keywords:** *Aeromonas*, Quorum sensing, Quorum sensing inhibition, Antimicrobial resistance, Horizontal gene transfer

## Abstract

**Abstract:**

*Aeromonas* species (spp.) are well-known fish pathogens, several of which have been recognized as emerging human pathogens. The organism is capable of causing a wide spectrum of diseases in humans, ranging from gastroenteritis, wound infections, and septicemia to devastating necrotizing fasciitis. The systemic form of infection is often fatal, particularly in patients with underlying chronic diseases. Indeed, recent trends demonstrate rising numbers of hospital-acquired *Aeromonas* infections, especially in immuno-compromised individuals. Additionally, *Aeromonas*-associated antibiotic resistance is an increasing challenge in combating both fish and human infections. The acquisition of antibiotic resistance is related to *Aeromonas*’ innate transformative properties including its ability to share plasmids and integron-related gene cassettes between species and with the environment. As a result, alternatives to antibiotic treatments are desperately needed. In that vein, many treatments have been proposed and studied extensively in the fish-farming industry, including treatments that target *Aeromonas* quorum sensing. In this review, we discuss current strategies targeting quorum sensing inhibition and propose that such studies empower the development of novel chemotherapeutic approaches to combat drug-resistant *Aeromonas* spp. infections in humans.

**Key points:**

*• Aeromonas notoriously acquires and maintains antimicrobial resistance, making treatment options limited.*

*• Quorum sensing is an essential virulence mechanism in Aeromonas infections.*

*• Inhibiting quorum sensing can be an effective strategy in combating Aeromonas infections in animals and humans.*

## Introduction

*Aeromonas* species (spp.) are ubiquitous in nature predominately found in freshwater habitats and estuarine ecosystems. The organism commonly infects fish, amphibians, and reptiles, wreaking havoc on the fish-farming industry. The first documented case of a human *Aeromonas* infection was recorded in 1951 when the organism was cultured from cerebral spinal fluid during a patient’s autopsy in Jamaica (Caselitz 1996). Since this landmark case, 36 spp. have been added to the genus, and at least 19 of them have been classified as emerging human pathogens (Fernández-Bravo and Figueras 2020). Unlike many other human pathogens, *Aeromonas* spp. are unique in their ability to inhabit an enormous range of hosts. In addition to humans, they have been isolated from leeches, insects, mollusks, birds, livestock, fresh produce, preserved food, domestic animals, drinking water, and wastewater sludge (Didugu et al. 2015; Govender et al. 2021; Janda and Abbott 2010; McMahon and Wilson 2001; Wang et al. 2011; Wu et al. 2019). The organism’s ability to grow at refrigeration temperatures is an added concern in the food industry (Hoel et al. 2019). Alarmingly, human *Aeromonas* infections are not associated with just one predictable tissue type or set of symptoms but rather have been implicated in an impressive array of clinical syndromes including wound/soft tissue infections, septicemia/bacteremia, gastroenteritis, colitis, intraabdominal infections/peritonitis, urinary tract infections, pneumonia, and even dreaded necrotizing fasciitis (Janda and Abbott 2010). Historically, symptomatic infections have mostly been associated with immuno-compromised patients, and a number of comorbidities are commonly correlated with severe infection, mostly liver disease (Clark and Chenoweth 2003; Valcarcel et al. 2021; Xu et al. 2022). While the majority of *Aeromonas* research is focused on aquaculture and relieving the economic burden of fish disease, there is a pressing need to understand this pathogen in a human-disease context and to innovate and develop novel, clinically relevant treatments as antimicrobial resistance (AMR) continues to spread in this pathogen.

Overuse/misuse of antibiotics, both clinically and commercially, has accelerated AMR development world-wide in a large number of human bacterial pathogens. This has resulted in bacterial infections that are increasingly difficult to treat, leading to higher mortality rates and longer hospital stays (Orosz et al. 2022; Wagenlehner and Dittmar 2022). The seemingly unchecked emergence of AMR pathogens poses a significant global threat to public health, prompting the need for a better understanding of the molecular mechanisms of resistance and the development of novel countermeasures. *Aeromonas* spp. are no exception to this emerging trend and have demonstrated their ability to rapidly acquire and share new AMR genes. Identifying/developing solutions to this problem requires a multifaceted approach including achieving a better understanding of *Aeromonas* spp. acquisition and retention of AMR genes, as well as identifying and characterizing virulence factors/mechanisms as potential drug targets. One promising antibiotic-alternative drug target is quorum sensing (QS), an essential virulence mechanism for *Aeromonas* spp. during human infections.

## Antibiotic resistance in *Aeromonas:* an ongoing problem

Considering *Aeromonas*’ pervasiveness in both ecological and clinical environments, promiscuity, and ability to cope with (and endure) environmental stressors, it should not be overlooked as a potentially significant source and/or reservoir of clinically relevant AMR genes. AMR mechanisms, particularly the presence of ß-lactams, including carbapenemases (Hayes et al. 1994, 1996), have been well characterized in *Aeromonas* spp. over the years. Chromosomally encoded ß-lactams were among the first to be detected and genetically characterized in *Aeromonas* (Iaconis and Sanders 1990; Ko et al. 1998; Walsh et al. 1997). Today, AMR genes that encode penicillinases, cephalosporinases, and metallo-ß-lactamases are of common occurrence in many *Aeromonas* spp. (Nwaiwu and Aduba 2020; Pourmohsen et al. 2023; Hilt et al. 2020). AMR genes do not observably accumulate in one species of *Aeromonas* more than another (Bertran et al. 2021). This is, in part, due to the fact that accurate identification of *Aeromonas* on a species level is historically problematic due to standard diagnostic techniques that are not tailored to *Aeromonas* spp. and shifting taxonomic definitions (Zhang et al. 2023; Fernández-Bravo and Figueras 2020). For example, *A*. *dhakensis* is being reevaluated in recent years for clinical significance, especially as a culprit in monomicrobial, systemic infections because of frequent misidentification as *A*. *hydrophila* (Wu et al. 2015, Puah et al. 2022). These factors combine to make studying species-specific AMR trends difficult.

Importantly, clinical AMR *Aeromonas* strains have been isolated across the globe; 3rd-generation cephalosporin-resistant *Aeromonas* has been isolated in Southern India (Bhaskar et al. 2015), and 3rd-generation cephalosporin and carbapenem-resistant isolates have been found in Croatia (Drk et al. 2023). Two broad-spectrum carbapenemase KPC-24-producing *A*. *veronii* strains were isolated recently from hospital sewage in China (Yang et al. 2022). Indeed, AMR profiles have been characterized in *Aeromonas* spp. isolated from India (Indra et al. 2015), including North Bengal (Dey Bhowmick and Bhattacharjee 2017), Tunisia (Bargui et al. 2023), Egypt (El-Hossary et al. 2023), Thailand (Hatrongjit et al. 2020), European countries, and Brazil, just to name a few (Table 1). Sequence analysis of nine independent *A*. *veronii* isolates from fish, humans, and Brazilian environments found all isolates to be remarkably similar to the uploaded *Aeromonas* genomes found in NCBI, demonstrating that widespread distribution of AMR genes in *Aeromonas* can originate from vastly different sources and geographic locations (Maia et al. 2023). Environmental isolates unassociated with human/clinical disease also demonstrate an alarmingly high rate of AMR. This suggests a possible ecological reservoir of AMR genes with the potential to be transferred to human-associated strains (Canellas et al. 2023; Goñi-Urriza et al. 2000; Igbinosa et al. 2015). It is clear that *Aeromonas* spp. are formidable enemies capable of horizontally spreading AMR genes, and it is important to understand how and why such propensity for AMR acquisition and retention exists.

**Table 1:** A table summarizing representative studies characterizing AMR in *Aeromonas* over the years

*Aeromonas* spp.	Method of spp. identification	Detected resistance	Method of resistance detection	Location	Reference
*A*. *caviae*, *A*. *sobria*, *A*. *hydrophila*	Biochemical assays	ß-Lactamases	Substrate profile assay, isoelectric focusing	USA	Bakken et al. (1988)
*A*. *hydrophila*, *A*. *sobria*	Biochemical assays	ß-Lactamases	Isoelectric focusing	USA	Iaconis and Sanders (1990)
*A*. *salmonicida subsp*. *achromogenes*	Unspecified	Three ß-lactamases	Anion and cation exchange chromatography	Scotland	Hayes et al. (1994)
*A*. *hydrophila*, *A caviae*	Unspecified	Three β-lactamases	Anion and cation exchange chromatography	Scotland	Hayes et al. (1996)
*A*. *caviae*, *A*. *veronii*, *A*. *hydrophila*	Unspecified	*ampS*, *cepS*, *cphA*	DNA probes	Italy, UK, Australia, France	Walsh et al. (1997)
*A*. *hydrophila*	16 s rRNA sequencing	Ampicillin – sulbactam, cephalothin, cefoxitin, cefmetazole, cefuroxime, cefotaxime	In vitro susceptibility (disk diffusion)	Taiwan	Ko et al. (1998)
*A*. *hydrophila*, *A*. *sobria*, *A*. *caviae*, *A*. *veronii*, *A*. *salmonicida*	Biochemical assays	*TetA*	DNA probes	England	Rhodes et al. (2000)
*A*. *caviae*, *A*. *sobria*, *A*. *hydrophila*	Biochemical assays	Nalidixic acid, tetracycline, Fosfomycin, tobramycin and cotrimoxazole, cefotaxime, chloramphenicol, gentamicin	In vitro susceptibility (disk diffusion)	Spain, France	Goñi-Urriza et al. (2000)
*A*. *caviae*	16 s rRNA sequencing, *gyrB* sequencing	*bla*_*TEM-24*_	Restriction profile comparison	France	Marchandin et al. (2003)
*A*. *caviae*	Unspecified	Nalidixic acid, ciprofloxacin, norfloxacin	In vitro susceptibility (disk diffusion)	India	Sinha et al. (2004)
*A*. *caviae*, *A*. *sobria*, *A*. *hydrophila*, *A*. *encheleia*, *A*. *veronii*	Unspecified	Ampicillin, chloramphenicol, kanamycin, cefazolin, nalidixic acid, sulphamethoxazole, streptomycin, trimethoprim–sulphamethoxazole, tetracycline	In vitro susceptibility (disk diffusion)	Taiwan	Chang et al. (2007)
*A*. *caviae*	16 s rRNA and *rpoB* sequencing	*bla*_*IMP-19*_	Isoelectric focusing	France	Neuwirth et al. (2007)
*A*. *hydrophila*	Unspecified	bla_*VIM-4*_	PCR/Sanger sequencing	Hungary	Libisch et al. (2008)
*Aeromonas spp*.	Unspecified	Ceftriaxone *bla*_*CTX-M*_, *AmpCBL*	In vitro susceptibility (disk diffusion) and PCR	India	Bhaskar et al. (2015)
*Aeromonas spp*.	Biochemical assays	Amoxicillin, aztreonam, and cephalothin *bla*_*TEM-1*_	In vitro susceptibility (disk diffusion) and PCR	India	Indra et al. (2015)
*Aeromonas spp*.	Morphological, cultural, and biochemical characterization	Clindamycin, oxacillin, trimethoprim, novobiocin, and ticarcillin *bla*_*TEM*_, bla_P1_	In vitro susceptibility (disk diffusion) and PCR	South Africa	Igbinosa et al. (2015)
*Aeromonas spp*.	Biochemical assays and 16 s rDNA amplification and sequencing	Ampicillin, amoxi-clav, cefazolin, cefotaxime, ceftazidime, cefuroxime, cephalothin, clindamycin, erythromycin, nalidixic acid, nitrofurantoin, norfloxacin, oxacillin, streptomycin, and ticarcillin	In vitro susceptibility (disk diffusion) and PCR	India	Dey Bhowmick and Bhattacharjee (2017)
*Aeromonas veronii*	MALDI-TOF* and WGS*	*mcr-3*.*41*, *bla*_*cphA3*_, *bla*_*OXA-12*_, *tetA*, *rsmA*, and *adeF*	Whole-genome sequence analysis	Thailand	Hatrongjit et al. (2020)
*A*. *veronii*	MALDI-TOF* and WGS*	bla_kpc-24_	PCR/Sanger sequencing	China	Yang et al. (2022)
*A*. *caviae*, *A*. *hydrophila*, *A*. *media*, *A*. *veronii*	MALDI-TOF* and 16 s rRNA sequencing	Amoxicillin, amoxi-clav, cephalexin, cefuroxime, ceftazidime, cefepime, ertapenem, imipenem, meropenem, and ciprofloxacin bla_KPC-2_, bla_NDM-1_, bla_VIM-2_, bla_OXA-48_, and bla_IMP-13_, *bla*_*GES-5*_, *bla*_*TEM-1*_, *bla*_*SHV-12*_, *bla*_*VEB-9*_, *bla*_*MOX*_, *bla*_*CIT*_, *bla*_*FOX*_, *bla*_*ACC*_	In vitro susceptibility and PCR/Sanger sequencing	Croatia	Drk et al. (2023)
*A*. *hydrophila*, *A*. *sobria*, *A*. *caviae*, *A*. *salmonicida*	Biochemical assays	Ampicillin, amoxi-clav, amikacin, chloramphenicol, cephalothin, cefuroxime, cefotaxime, cefepime, aztreonam, imipenem, gentamicin, ciprofloxacin, polymyxin B	In vitro susceptibility (disk diffusion)	Tunisia	Bargui et al. (2023)
*Aeromonas spp*., *A*. *hydrophila*	Biochemical assays	Ceftriaxone, cefotaxime, ceftazidime, cefixime, tobramycin, kanamycin, streptomycin, tetracycline, ciprofloxacin, norfloxacin, nalidixic acid, chloramphenicol	In vitro susceptibility (disk diffusion)	Egypt	El-Hossary et al. (2023)
*A*. *veronii*	WGS	*vat*, *cphA*, *bla*_*OXA*_, *dfr*, *mcr-3*, *mcr-7*.*1*, *sul*, efflux pumps	WGS analysis	Brazil	Maia et al. (2023)
*A*. *jandaei*, *A*. *veronii*, *A*. *caviae*, *A*. *sanarellii*, *A*. *hydrophila*, *A*. *molluscorum*	16 s rRNA sequencing	Cefotaxime, imipenem, amoxi-clav, ciprofloxacin, ceftazidime, tetracycline, trimethoprim-sulfamethoxazole *bla*_*TEM*_, *bla*_*KPC*_, *mcr-3*,	In vitro susceptibility and PCR	Brazil	Canellas et al. (2023)
*A*. *veronii*	MALDI-TOF* and 16 s rRNA sequencing	Cephamycin, cephalosporin, amoxicillin, tetracycline *cphA4*, *vatF mcr-7*.*1*, *bla*_*FOX-7*_, *bla*_*OXA-12*_, *tetE*, *tetR*	In vitro susceptibility (disk diffusion) and WGS analysis	USA	Dubey et al. (2023)
*Aeromonas spp*.	Vitek-2 and MALDI-TOF*	Ampicillin, ciprofloxacin, florfenicol, chloramphenicol, tetracycline, trimethoprim-sulfamethoxazole, gentamicin, colistin *bla*_*KPC-2*_, *bla*_*NDM-1*_, *bla*_*VIM-1*_* mcr-3*, *tmexCD-toprJ*	In vitro susceptibility (microdilution) and WGS analysis	China	Wu et al. (2023)

Included are studies describing emerging AMR genes or trends in *Aeromonas* (both clinical or environmental) which were unique for the given year or geographical region during the time of publication. AMR can be studied and tracked in several ways including phenotypic susceptibility testing, AMR gene identification, and antibiotic class resistance detection. Therefore, the table includes spp. identified in the study (if an attempt was made to do so), the spp. identification method, the resistance that was characterized in the study, the methodology it was characterized with, and the global location.

*MALDI-TOF, Matrix-assisted laser desorption/ionization – time-of-flight; *WGS*, whole-genome sequencing

## Sources and selective pressures for AMR acquisition

As alluded to earlier, one commonly studied mechanism of AMR acquisition in *Aeromonas* is horizontal gene transfer from other bacteria. *A*. *caviae*, for example, has been shown to be naturally competent and readily acquires DNA from its environment (Sayeed et al. 1996). In one study, 73% of environmental *Aeromonas* isolates were able to serve as recipients of donor DNA, while 100% of tested isolates were able to act as donors to at least some other aeromonads under optimal laboratory conditions (Tris buffer with magnesium or calcium, pH 5–8, and a saturating concentration of 0.5 μg of DNA per assay, at 30 °C; sodium was also required) (Huddleston et al. 2013). On account of this naturally transformative state, it is no surprise that so many AMR genes have been found in *Aeromonas* spp. that are derived from other common but unrelated pathogens. For example, a Verona integron-encoded family metallo-β-lactamase (VIM) producing *A*. *hydrophila* strain carrying a *VIM-4* gene was described in a case report from Budapest in 2008. Sequencing showed an identical match to a previously characterized integron in *Pseudomonas aeruginosa* from southern Hungary, suggesting DNA transfer between the two (Libisch et al. 2008). Temoniera-24 (*TEM-24*), a prominent extended-spectrum β-lactamase gene variant, was observed in a clinical isolate of *Aeromonas* for the first time in 2003. *TEM-24* is typically isolated across Western Europe from *Enterobacter* spp. and *Pseudomonas* spp., and since this particular *Aeromonas* spp. was isolated alongside *Enterobacter aerogenes*, this also suggests cross-species horizontal AMR gene acquisition (Marchandin et al. 2003).

Another mechanism that could be promoting the alarming rate of AMR acquisition is the presence of AMR genes in the context of mobile genetic elements (MGE) such as plasmids and integrons. *Aeromonas* spp. are known to possess a collection of plasmids constituting its plasmidome. The *Aeromonas* plasmidome is of particular interest in the context of AMR genes and other virulence factors (Vincent et al. 2021). Virulence-related plasmids released by bacterial cells have been shown to persist in harsh environments such as treated wastewater and can readily be acquired by nearby pathogens (Drk et al. 2023). Additionally, integrons, small sections of chromosome that can capture gene cassettes from the environment and incorporate them into the genome via integrase genes and site-specific recombination, have been found to play an important role in the acquisition and spread of antibiotic resistance genes (Fluit and Schmitz 1999). Such integrons have been found within multiple species of the *Aeromonas* genus. Characterization of 133 *Aeromonas* spp. isolates (50 *A*. *caviae*, 45 *A*. *hydrophila*, 31 *A*. *sobria*, 6 *A*. *encheleia*, and 1 *A*. *veronii*) revealed the presence of several different class I integrons, including 10 different gene cassettes, encoding resistance to a variety of antibiotics including trimethoprim, aminoglycosides, ß-lactams, and phenicol. As to be expected, antibiotic resistance rates were different between integron-positive and integron-negative strains. Specifically, resistance to trimethoprim and trimethoprim–sulphamethoxazole was more commonly associated with integron-positive isolates, and all integron-positive isolates were resistant to more than 3 antibiotics. In fact, resistance to as many as 10 antimicrobial chemotherapeutics was observed in some integron-positive strains (Chang et al. 2007). Additionally, a global study of 38 *A*. *salmonicida* isolates revealed that 21/38 isolates contained a class I integron with all gene cassettes described in the study being associated with human bacterial infections (L'Abée-Lund and Sørum 2001).

Non-pathogenic, environmental/aquatic *Aeromonas* spp. have also proven to be significant sources/reservoirs of AMR gene acquisition of clinically relevant strains. In one such example, the transfer of oxytetracycline-resistant plasmids between *Aeromonas* spp. found in fish hatcheries and *Aeromonas* spp. recovered from hospital effluent was observed in *A*. *hydrophila* (8 isolates), *A*. *sobria* (6 isolates), and *A*. *caviae* (1 isolate) (Rhodes et al. 2000). Additionally, *A*. *veronii* isolated from catfish ponds in the South-Eastern USA was found to harbor tetracycline resistance gene on a MGE, similar to ones found in *Vibrio parahaemolyticus* and other *Aeromonas* spp. isolated from human stool (Dubey et al. 2023). When 66 *Aeromonas* isolates [*A*. *caviae* (58%), *A*. *hydrophila* (17%), *A*. *media* (11%), and *A*. *veronii* (11%)] from both untreated hospital wastewater and treated municipal water were examined, almost all of them (65/66) demonstrated multidrug-resistant phenotypes. Prevalent carbapenem genes found among the isolates included *bla*_*KPC-2*_, *bla*_*VIM-2*_, *bla*_*OXA-48*_, and *bla*_*IMP-13*_, with the latter three being described for the first time in *Aeromonas*. This same study demonstrated the ability of some of these *Aeromonas* isolates to transfer these resistance phenotypes to susceptible recipients (*Escherichia coli*), suggesting conventionally treated municipal and untreated hospital wastewater may be a reservoir for AMR, and that *Aeromonas* spp. could be mediating the spread of AMR to other pathogens in that environment (Drk et al. 2023). By demonstrating overlap between aquatic and clinical *Aeromonas* spp., these findings warn of the perils of compartmentalizing human and agricultural/environmental niches and conversely suggest that they should be considered as one combined environment since the transfer of genetic information can occur between them (Jones et al. 2023). Roh and Kannimuthu (2023) in a recent genomic analysis of the resistomes of 400 *Aeromonas* aquaculture strains found resistance against carbapenem, fluoroquinolone, cephalosporin, elfamycin, aminoglycoside, and tetracycline was “more or less evenly distributed across all species, while resistance against the other classes varied between species” (Roh and Kannimuthu 2023). This distribution highlights the genetic promiscuity displayed by *Aeromonas* across species and underscores its potentially global relevance as an indicator organism of the spread of antibiotic resistance (Usui et al. 2016).

## Mechanisms of *Aeromonas*-associated drug resistance

*Aeromonas* employs a multivariate platform of strategies that confer antimicrobial resistance. One such strategy is through the exploitation of escape mutations in genes encoding the protein targets of the antibiotics. For example, quinolone resistance observed in an *A*. *caviae* human isolate was a result of an accumulation of point mutations in the type II topoisomerase genes *gyrA* and *parC* allowing for the continued function of the enzymes while reducing antibiotic binding affinity (Sinha et al. 2004). Another AMR mechanism employed by *Aeromonas* is the use of substrate-specific antibiotic degrading enzymes such as ß-lactamases (Majiduddin et al. 2002; Rasmussen and Bush 1997). For example, *MOX-9*, a class C enzyme belonging to a novel sub-lineage of MOX ß-lactamases, was found to be encoded by a chromosomal transposon in *A*. *media*. Biochemical characterization of this *MOX-9* gene revealed a strong binding preference for cephalosporins and cephamycins. By comparing MOX-9 binding affinity and its hydrolysis activity to other more common MOX-type enzymes, this study not only demonstrated the variations that exist within this family of resistance genes but also provided a genetic context by which resistance genes can be easily mobilized onto transmissible plasmids and horizontally shared among other organisms (Piccirilli et al. 2022).

A third strategy is the use of broad-spectrum, non-specific AMR techniques such as drug uptake resistance, efflux pumps, and/or enhanced biofilm production. In one demonstration, when the *ompR* gene encoding an outer membrane protein (OMP) was deleted in an *A*. *veronii* isolate, increased sensitivity of the mutant culture to both ceftriaxone and neomycin, two different classes of drugs, was observed. Interestingly, the Δ*ompR* mutant was shown to exhibit reduced biofilm production as well. It was speculated that this increased antibiotic susceptibility may be due, in part, to the reduction in biofilm formation since biofilm typically impedes an antibiotic’s access to the bacteria (Wang et al. 2023b). Indeed, when 29 OMP knockout strains were created in *A*. *hydrophila*, 22 gene deletions affected susceptibility levels to at least one of the 20 antibiotics tested. That being said, no OMP mutant exhibited consistent responses to all the tested antibiotics, eluding to more complicated downstream signaling/regulatory mechanisms underlying OMP-related drug uptake (Li et al. 2019). Additionally, when the porin protein Aha1 was mutated in *A*. *hydrophila* at its lysine-acetylation sites, increased resistance to tetracyclines and ß-lactams was observed, presumably due to decreased drug uptake (Zhang et al. 2022d). Recently, in China, a resistance-nodulation-division (RND)-type efflux pump gene cluster named *tmexCD1-toprJ1636* was discovered in a related Gram-negative enteric pathogen *Klebsiella pnuemoniae* which confers resistance to different classes of antibiotics including tetracyclines, cephalosporins, aminoglycosides, phenicol, quinolones, and the last-resort antibiotic tigecycline (Lv et al. 2020). Such an efflux pump has since been shown to play a role in *Aeromonas* spp. drug resistance as well; in an environmental study, 36 of the 636 *Aeromonas* spp. isolated from livestock, meat, water, and humans [*A*. *caviae* ( 22), *A*. *hydrophila* (5), *A*. *salmonicida* (1), and *A*. *veronii* ( 8)] were positive for the multidrug-resistant gene cluster mentioned above, either encoded chromosomally or on a plasmid. Importantly, the characterized *tmexCD-toprJ* genes were associated with different *Aeromonas* spp., phylogenetic lineages, environments, and genetic locations and were surrounded by varying MGEs, demonstrating alarming diversity (Wu et al. 2023).

In reality, AMR in *Aeromonas* likely results from many different complicated factors, all playing simultaneous and even interactive roles. Employing a proteomic approach, a quinolone, norfloxacin (NOR), stress response study in *A*. *hydrophila* revealed 186 downregulated proteins and 220 upregulated proteins following exposure. Interestingly, many of the differentially expressed proteins were involved in sulfur metabolism and homologous recombination. Seven of these differentially expressed proteins were chosen as targets for site-directed mutagenesis in their encoding genes. Some mutants exhibited increased sensitivity to NOR such as ΔAHA_0904 (an uncharacterized protein) and Δ*cirA* (colicin I receptor), whereas the Δ*hlyD* (in the secretion family) mutant significantly increased NOR resistance. Other mutants, ΔAHA_4275 (a ferrichrome receptor), Δ*icd* (isocitrate dehydrogenase [NADP]), Δ*cheV* (chemotaxis coupling protein), and Δ*ppsA* (phosphoenolpyruvate synthase) displayed no differences compared to the parental *A*. *hydrophila* strain (Liu et al. 2023). This suggests that genes with no apparent ties to AMR can play an important role in an organism’s resistance/susceptibility to antibiotic stress.

Transcriptional regulators have also been shown to influence *Aeromonas* spp. drug resistance. More specifically, the transcriptional regulator AhslyA was shown to play a role in fluoroquinolone resistance. In an *A*. *hydrophila* Δ*ahslyA* mutant, increased fluoroquinolone Enoxacin (ENX) sensitivity was observed. Proteomic analysis revealed differentially produced proteins involved in DNA metabolism, the SOS response, and cell communication following ENX treatment. Site-specific mutations were then engineered in several targets’ encoding genes, three genes related to decreasing protein abundance (AHA_0655, AHA_1195, and AHA_3721), and three genes related to increasing protein abundance (AHA_1239, AHA_2114, and *narQ*). The ΔAHA_2114 and Δ*narQ* mutants had slightly decreased resistance to ENX at 0.01 μg/mL, and mutants ΔAHA_1239 and ΔAHA_3721 demonstrated an increase in resistance to ENX at 0.01 μg/mL. This further demonstrates the genetic diversity of expression/regulation involved in conferring drug resistance (Li et al. 2021). Collectively, these studies underscore the complex network of overlapping known pathways and mechanisms involved in AMR. When considering the potential contributions of yet unknown pathways, the network becomes even more complex.

## Quorum sensing

**Fig. 1:**
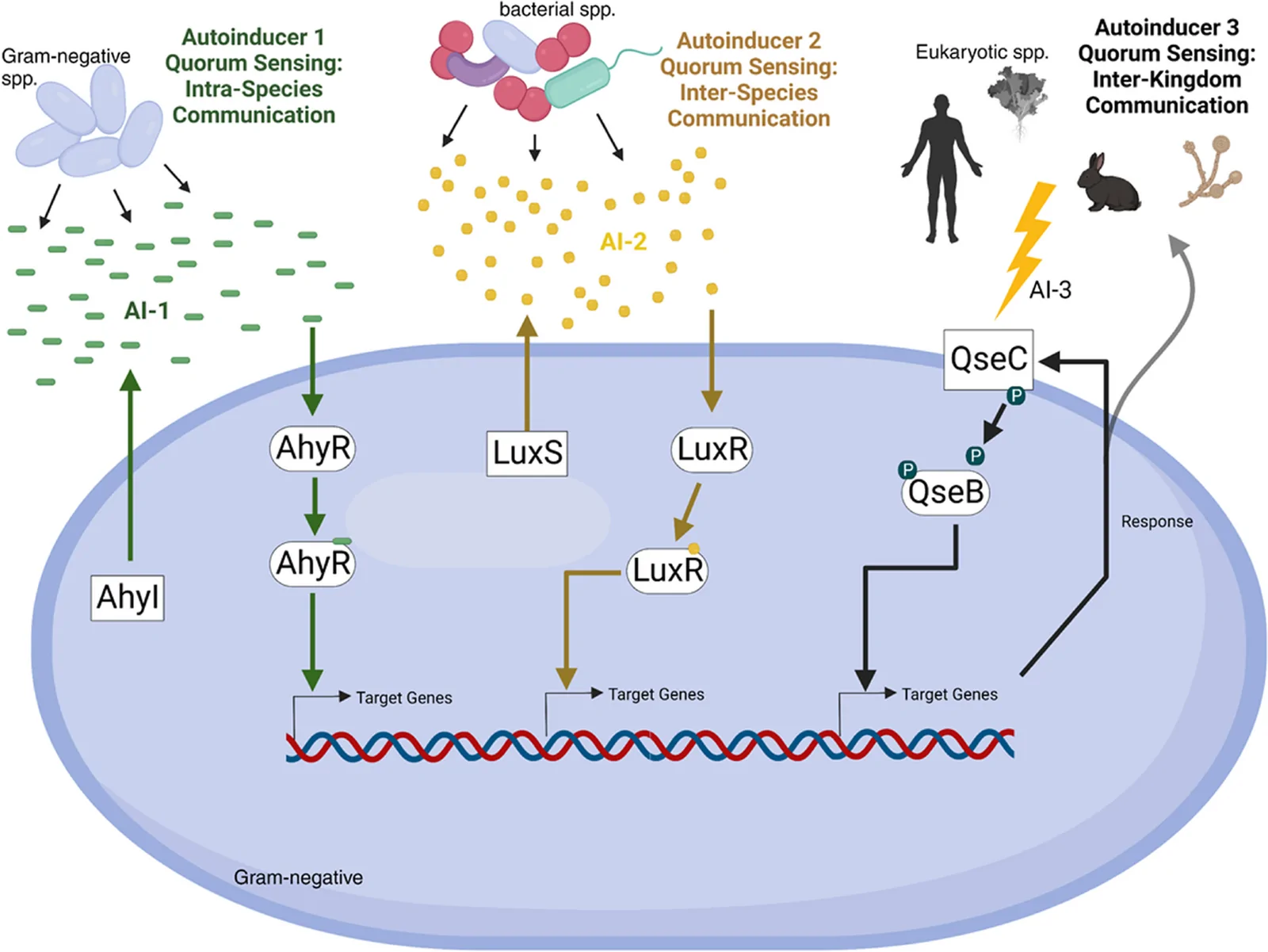
A schematic demonstrating the basic mechanisms that govern the three QS systems: AI-1 QS (far left) is only found in Gram-negative bacteria and is thought to be the mode of intra-species communication. Bacteria in a community simultaneously produce AI-1 signal via an AI-1 synthase (AhyI) where the signal is then sensed and responded to via the response regulator AhyR. AI-2 QS (center) is found in both Gram-positive and Gram-negative bacteria and is thought to be the mechanism of communication between different bacterial species. AI-2 signal is produced by all members of the bacterial community via LuxS. LuxR is responsible for sensing and responding to AI-2 signals. AI-3 QS (far right) is a two-component phosphorylative response system thought to be a mode of communication between prokaryotes and eukaryotes (inter-kingdom). Signals produced by a eukaryotic host collectively known as AI-3 cause a conformational change in membrane-bound sensor kinase (QseC), allowing for phosphorylation of the cytoplasmic response regulator (QseB), which activates it. (Image produced in BioRender)

Quorum sensing (QS), broadly, is a sophisticated mechanism of communication utilized by bacteria to coordinate behavior in a population. There are three major types of quorum sensing systems in *Aeromonas* known as autoinducers 1, 2, and 3. Autoinducer 1 (AI-1) QS is a system found exclusively in Gram-negative bacteria and is thought to detect and respond to the population density of members of the same species in an environment (Vanetti et al. 2020). Autoinducer 2 (AI-2) QS is a mechanism thought to mediate cross-species communication, given the machinery for this system is found in both Gram-positive and Gram-negative bacteria (Zhao et al. 2018). Autoinducer 3 (AI-3) QS is a two-component response system found in bacteria that responds to signals produced by members of the eukaryotic kingdom, demonstrating its use as an inter-kingdom mode of communication (Fan et al. 2022) (Fig. 1).

In many pathogenic bacteria, including *Aeromonas* spp., QS has been shown to globally regulate virulence gene expression and/or disease-causing mechanisms. Some of these virulence factors/mechanisms, utilized by *Aeromonas* and regulated by QS, include biofilm formation, motility, and effector protein secretion through various (e.g., types 2, 3, and 6) secretion systems (Table 2).

**Table 2:** Well-characterized *Aeromonas* virulence factors and their respective functions including helpful references on the topic

Virulence factor	Description	Function	Reference
Extracellular enzymes/toxins			
*exoA*	Exotoxin A	A toxin that modifies eukaryotic ribosomal elongation factor 2 which results in inhibition of protein synthesis and host cell death	Fernández-Bravo et al. (2019); Masuyer (2020)
*act*	Cytotoxic enterotoxin	T2SS-associated enterotoxin. Activates proinflammatory cytokine production in macrophages. Elevates the production of cyclic AMP in epithelial cells, possesses hemolytic and cytotoxic activities	Chopra et al. (2000); Rather et al. (2019)
*alt*	Heat labile cytotonic enterotoxin	Promotes the degeneration of villi and crypts of the small intestine	Rather et al. (2019; Sierra et al. (2011)
*ast*	Heat-stable cytotonic enterotoxin	Stimulates fluid secretion in the small intestine by increasing cAMP levels in mucosal cells	Rather et al. (2019; Sierra et al. (2011)
*ahyB*	Protease	Degrades host azocasein and has elastolytic activity	Cascón et al. (2000)
*aerA*	Aerolysin	Pore-forming toxin. Pores disrupt the host cell’s osmotic balance leading to lysis and tissue damage	Ran et al. (2018)
*hlyA*	Hemolysin	Hemolysis of blood cells	Wang et al. (2003)
*pro*	Protease	Promotes invasion and nutrient scavenging by directly damaging host tissue. Proteolytic activation of toxins. Promotes evasion of initial host defenses by inactivating the complement system	Bhattacherjee et al. (2021); Sakai (1985); Shao et al. (2023)
*ela*	Elastase	Allows for evasion of immune defenses by cleaving IgA. Stimulates alginate synthesis	Barger et al. (2021)
*lip*	Lipase	Generation of free fatty acid through lipolytic activity. Impairs host immune function	Gurkok and Ozdal (2021); Papulzai et al. (2020)
*eno*	Enolase	Glycolytic enzyme which binds to human plasminogen on cell surfaces leading to cancerous conditions, neurological disease, and autoimmunity	Sha et al. (2003)
*ser/ahp*	Serine protease	Aids in invasion by breaking down host proteins. Evasion of the immune system by deactivating specific components	Feng et al. (2022); Ueda et al. (2022)
Structural components			
Flagella	Polar and lateral	Plays roles in biofilm formation, motility, and enterocyte adhesion, and promotes colonization and invasion	Cheng et al. (2023); Lau et al. (2023)
A-layer	Outer layer protein array	An array of tetragonal proteins, known as A-protein, which is tethered to the cell by LPS. Provides protection against immune defenses such as serum-mediated killing and macrophage cytotoxicity	Mendoza-Barberá et al. (2021); Paquet et al. (2019)
Capsule	Outer layer of polysaccharides	Aids in host colonization and invasion; prevents phagocytosis by host macrophages	Heiss et al. (2019); Mendoza-Barberá et al. (2021)
Fimbriae/Pilli		Filamentous structures on the bacterial surface that aid in adhesion. Have been implicated in other functions, such as phage binding, DNA transfer, biofilm formation, cell aggregation, host cell invasion, and twitching motility	Garg et al. (2023)
Non-filamentous Adhesins	Outer membrane proteins/porins	Promote binding of the bacteria to carbohydrate-rich surfaces like erythrocytes and intestinal human cells	Ebanks et al. (2005)
Secretion systems/effectors			
T3SS	Structural components that result in effector secretion	A complex bacterial machine that transports small proteins directly from the bacterial cytoplasm across the inner and outer bacterial membrane to the extracellular environment or directly into the target host cell	Pillai (2006)
*aexT/aexU*	T3SS effector protein	A T3SS effector, homologous to the *P*. *aeruginosa* effectors ExoT/ExoS, with ADP-ribosyltransferase and GTPase activities	Braun et al. (2002); Burr et al. (2003); Sha et al. (2007)
*aopP* and other T3SS effector proteins	T3SS effector protein	Interferes with host cell signaling and in other ways modifies/regulates pathogenicity	Matys et al. (2020); Rangel et al. (2019); Zhang et al. (2022a)
T6SS	Structural Components that result in effector secretion	A bacterial machine that constitutes a phage-like injectosome complex that serves to translocate effectors directly into competing bacterial cells as well as eukaryotic host cells	Suarez et al. (2008)
*hcp*	Hemolysin coregulated protein	Surface-exposed structural components that might be released in culture supernatants or into eukaryotic cells during translocation	Cai et al. (2022); Tekedar et al. (2018)
*vgrG*	Valine-glycine repeat protein G	Acts as a conduit for T6SS effector proteins to be transported from the bacterial cytoplasm into the target cell. Can be involved in T6SS expression regulation	Tekedar et al. (2018)
*evpP/*T6SS-associated effectors	Secreted T6SS effector proteins	Various functions depending on the specific effector; de-ubiquitinase activity, carries a payload of toxic proteins/enzymes that disrupt cell function, degrades cell components, and modifies the environment to be more favorable	Matys et al. (2020); Wang et al. (2023a)

### Autoinducer 1 quorum sensing: AhyRI

A QS system homologous to the LuxRI system in *V*. *fischeri* (Kempner and Hanson 1968) was first described in *Aeromonas* in 1997 (Swift et al. 1997). The system produces acyl-homoserine lactones (AHLs), molecular signals collectively known as AI-1, which are synthesized by the AHL synthase AhyI. A corresponding response regulator, AhyR, is then modulated by this signal (Chu et al. 2013; Swift et al. 1999; Van Houdt et al. 2007) to alter downstream gene expression. By far the most studied of the 3 *Aeromonas*-associated QS systems, AI-1 QS is ubiquitous across *Aeromonas* spp. (Jangid et al. 2007) and has been shown to influence the development of biofilm (Lynch et al. 2002), exo-proteases production (Khajanchi et al. 2009; Swift et al. 1999), outer membrane protein profiles, S (surface)-layer thickness (Bi et al. 2007), and type 6 secretion system (T6SS) effector secretion (Khajanchi et al. 2009) (Table 2). It has also been shown that mutations in this QS system result in decreased virulence potential of *Aeromonas*. More specifically, the virulence of a Δ*ahyR*Δ*ahyI* double mutant was reduced by 50% when compared to its parental strain *A*. *hydrophila* SSU [since re-classified as *A*. *dhakensis* (Grim et al. 2014)] in a murine model of infection (Khajanchi et al. 2009). In a fish infection model using a challenge dose of 10^9^ colony forming units (CFU)/ml, *A*. *hydrophila* J-1 mutant Δ*ahyR* was rendered avirulent, as evidenced by the 100% survival of challenged fish. In contrast, 100% fish mortality was observed when challenged with the parental strain at the same dose (Bi et al. 2007). To better understand the AI-1 QS pathway, an AHL lactonase was used to block AI-1 signaling. Further evaluation of the data revealed differential expression of genes, post AHL lactonase treatment, that were involved in a myriad of metabolic pathways including metabolite transport, amino acid metabolism, central metabolism, and respiration, suggesting universal metabolic regulation by AI-1 QS (Gui et al. 2017).

While it is well established that AI-1 QS plays a crucial role in the virulence of *Aeromonas* (Bi et al. 2007; Khajanchi et al. 2009), little is known about the specifics of AHL synthesis or substrate specificity. It was historically thought *Aeromonas* only had the ability to synthesize two AHLs, N-butanoyl-L-homoserine lactone (C4-HSL) and N-hexanoyl-L-homoserine lactone (C6-HSL) (Kirke et al. 2004; Swift et al. 1997, 1999). To expand that knowledge, one study successfully purified 6 unique AHLs from *A*. *hydrophila*, although C4-HSL and C6-HSL continued to be the most abundant signals. The mechanism by which AhyI is able to catalyze the formation of the various AHLs was proposed to likely employ small molecules S-adenosyl-L-methionine (SAM) and butyryl-acyl carrier protein (ACP) as facilitators. If indeed SAM and ACP are involved in AHL synthesis, then AHL synthesis utilizes an acyl-ACP-derived fatty-acyl substrate and not acyl-CoA, as previously thought (Jin et al. 2020).

### Autoinducer 2 quorum sensing: LuxS

The LuxS universal QS system mediated by AI-2 has also been described in the *Aeromonas* genus as early as 2008 (Kozlova et al. 2008). Unlike AI-1, this QS system is found in both Gram-positive and Gram-negative bacteria and is thought to be the means of cross-species communication (Xavier and Bassler 2003). Since its discovery, other publications have corroborated the existence of AI-2 systems in *Aeromonas* (Zhao et al. 2015); however, less research has focused on this QS system in *Aeromonas* spp. compared with AI-1 QS. The phenomenon generally observed has been an overall increase in virulence when AI-2 (*luxS* gene) is deleted. This is in sharp contrast to the deletion of the AI-1 system components. More specifically, an *A*. *dhakensis* SSU Δ*luxS* mutant exhibited decreased motility, increased virulence (as observed by increased lethality in a murine model), and altered biofilm structure. Surprisingly, the increased virulence in a septic mouse model of infection was not due to alterations/enhancements in hemolytic activity, AexU (a type 3 section system effector) translocation, or T6SS effector translocation (Kozlova et al. 2008) (Table 2). Furthermore, LuxS deficiency negatively affected expression levels of the A-layer gene encoding VapA, potentially reducing survivability in host macrophages (Meng et al. 2017). In an effort to uncover the mechanism(s) for these phenotypes, the DNA adenine-methyltransferase (Dam) encoding gene was overexpressed in both the parental and the Δ*luxS* mutant. The overexpression of *dam* caused the Δ*luxS* mutant to become hyper-motile and demonstrated increased hemolytic activity as compared to the isogenic *dam*-overexpressing parental strain. However, the overexpression of *dam* did not alter the virulence potential of the Δ*luxS* mutant in vivo. Taken together, these results suggest that the methylation of LuxS may play a role in the regulation of the AI-2 QS system (Kozlova et al. 2008) and needs further investigation.

To gain more insight into the signaling pathway downstream of AI-2 QS, the LuxS-regulated gene B protein (LsrB) was investigated in *A*. *veronii*. This protein belongs to the high-affinity substrate-binding protein family and is one of the two D-type receptors (LuxP and LsrB) of the AI-2 molecule in the AI-2 QS system. The major role of LsrB is to internalize extracellular AI-2 (Reading and Sperandio 2006). When this receptor was deleted in *A*. *veronii*, there was no apparent impact on growth, hemolytic activity, or antibiotic sensitivity. Motility was slightly decreased, likely on account of reduced flagellar gene expression, and a significant reduction in biofilm formation was also observed. Interestingly, the subsequent interruption of the AI-2 signaling pathway following LsrB deletion resulted in an unexpected decrease in virulence in a zebrafish model (as measured by an increased LD_50_) (Gao et al. 2023) contradicting the previous murine study (Kozlova et al. 2008). Additionally, AI-2 QS signaling seems to be affected by post-translation modifications (PTMs). By mapping out lysine-acetylation and lysine-succinylation sites in *A*. *hydrophila*, the sites were found to be largely overlapping. One such overlap was in the amino acid K165 in the *luxS* gene. Acetylated LuxS was found to negatively regulate LuxS enzymatic activity in *A*. *hydrophila*, while conversely, succinylated LuxS (at the same residue) positively regulated enzymatic activity. Interestingly, two distinct PTMs of LuxS on a specific residue oppositely influenced bacterial AI-2 QS activity (Sun et al. 2019), suggesting that the role LuxS plays in *Aeromonas*’ biological functions may be partially dependent on PTM status. This aspect may potentially contribute to the difference in phenotypic virulence in murine versus Zebra fish models and requires further investigation.

### Autoinducer 3 quorum sensing: QseB/QseC

A third QS system mediated by two-component regulatory proteins QseB and QseC, which respond to AI-3 molecules, was identified in *A*. *dhakensis* SSU in 2012 (Khajanchi et al. 2012). Since then, 15 environmental *Aeromonas* isolates from China have been found to possess *qseBC* genes, demonstrating the widespread nature of this system within the genus (Sarkodie et al. 2019). Of the three QS systems identified in *Aeromonas* spp., the QseB/QseC system is the most poorly understood. Upon discovery, a Δ*qseB* mutant was constructed in *A*. *dhakensis* SSU, and the mutant exhibited diminished swarming and swimming motility, increased biofilm density, reduced protease production, and a slightly decreased virulence with 30% lower mortality over a test period of 16 days in an in vivo murine model of septicemic infection compared to the parental strain (Khajanchi et al. 2012). In contradiction to this study, a recent 2023 study reported that AI-3 QS component deletions in *A*. *hydrophila* did not affect motility, decreased biofilm production, and promoted increased virulence in an in vivo fish model (Qin et al. 2023a). Given the contrary nature of these two reports in different animal models, clearly, more studies are needed to better understand this complicated QS system.

One interesting finding in a fish model of infection study (Qin et al. 2023a) was its use of the host-derived stress hormone norepinephrine (NE). QseBC has previously been shown to enable many entero-bacteria to sense and interact with the host-derived environment (Lustri et al. 2017; Moreira and Sperandio 2016). This may also be true for *Aeromonas* spp. since the addition of NE to the medium of *A*. *hydrophila* increased its growth rate and dramatically increased biofilm production. However, Δ*qseB* and Δ*qseC* mutants did not display the aforementioned NE-mediated responses, suggesting that host signaling molecules are, in some way, associated with *Aeromonas* behavior, likely through the AI-3 QS (Qin et al. 2023a).

## Interactions between QS systems

To complicate matters further, the three QS systems described above could possibly interact with one another, creating a complicated network of QS pathways replete with crosstalk and overlap. In an attempt to elucidate the ambiguity, one study systematically compared QS-related gene expression in mutants of all three QS systems. It was found that individual component deletions resulted in altered expression levels of the other QS system genes. In the Δ*ahyRI* mutant, *qseB*, *qseC*, and *luxS* genes were all upregulated. In the Δ*qseB* mutant, *ahyR* and *ahyI* gene expression levels were downregulated; however, no changes were observed in *luxS* expression. Finally, in the Δ*luxS* mutant, no changes were observed in *qseB* and *qseC* expression levels. Taken together, these findings demonstrate that crosstalk and/or compensatory interactions between/among the various *Aeromonas* spp. QS systems occur (Kozlova et al. 2012).

### Role of C-di-GMP in QS system interactions

C-di-GMP is a small signaling molecule that plays a crucial role in the regulation of bacterial behavior and physiology including all three *Aeromonas* spp. QS systems. An initial report demonstrated a link between c-di-GMP and AI-1 QS in *A*. *sobria* (Rahman et al. 2007). C-di-GMP overexpression in *A*. *hydrophila* was shown to enhance biofilm formation and reduce motility in the Δ*luxS* mutant and its parental strain. In contrast, the Δ*ahyRI* mutant only showed a marginal increase in biofilm formation with no effect on motility when c-di-GMP was overexpressed (Kozlova et al. 2011). Overexpression of c-di-GMP reduced protease activity in the Δ*qseB* mutant when compared to the isogenic parental strain, and no changes in protease activity in the Δ*ahyRI* mutant were observed. Furthermore, increased c-di-GMP expression in parental *A*. *dhakensis* SSU produced denser biofilms while increased c-di-GMP in the Δ*qseB* mutant decreased biofilm density (Kozlova et al. 2012). Collectively, the varying regulations each QS system exerts on one another, either positively or negatively, may be mediated by this small signaling molecule that has the demonstrable ability to communicate with all three.

Investigating the role of c-di-GMP in QS regulation led to the discovery that both AI-1 and AI-2 QS systems in *Aeromonas* affect expression levels of the transcriptional regulator LitR (Kozlova et al. 2011). The LitR homolog, HapR, has been shown to universally regulate virulence factors in *V*. *cholerae* (Kovacikova and Skorupski 2002). LitR has since been shown to bind to the promoter regions of the hemolysin and serine protease genes, as well as T6SS effector protein VrgG in *A*. *hydrophila*. LitR was also found to positively regulate hemolytic and extracellular protease activities (Zhao et al. 2023). This establishes LitR as a master transcriptional regulator used to control the expression of many essential virulence factors, and the expression level of LitR is regulated by both AI-1 and AI-2 QS systems, further demonstrating the overlap between the systems.

## QS inhibition: an alternative therapeutic to antibiotics

Understanding the *Aeromonas* spp. QS systems and their crosstalk could enable exploitations that may result in promising alternative therapeutics for *Aeromonas* infections and beyond. The need for alternative therapeutics is especially apparent in *Aeromonas* spp. on account of their being both the cause of severe infections in humans and reservoirs of AMR genes. In that vein, the first use of a QS inhibitor in *Aeromonas* appears in 2009 when Truchado et al. (2009) found culturing *A*. *hydrophila* with chestnut honey resulted in the degradation of AHLs and decreased biofilm production. Since then, many natural and synthetic compounds have been shown to decrease QS-mediated virulence factors including biofilm, motility, protease production, and hemolysis to great effect via QS inhibition in *Aeromonas* (Table 3). AI-1 QS inhibitor cinnamaldehyde was shown to significantly decrease virulence phenotypes of *A*. *hydrophila* (Li et al. 2023). The plant-derived citrus flavonoid, hesperidin methyl chalcone (HMC), was found to not only downregulate the QS gene *ahyR* but also reduce the overall virulence potential of *A*. *hydrophila* in an in vivo fish model (Roshni et al. 2023). Tannic acid has been proven to be an effective QS inhibitor in *A*. *hydrophila* with demonstrably lower expression levels of *ahyI* and *ahyR* post-treatment and reduced hemolysis, motility, and biofilm formation. Tannic acid treatment also resulted in decreased virulence potential in an in vivo fish model (Patel et al. 2017). Genistein caused the downregulation of *ahyRI* expression levels, decreased virulence factors like biofilm and aerolysin production, and increased survival in an in vivo fish model (Dong et al. 2021). Another compound, carvacrol, a naturally derived monoterpenoid present in many herbs, was found to decrease the virulence potential of *A*. *hydrophila* by inducing decreased biofilm formation, protease production, hemolytic activity, and AHL production. The transcriptional analysis uncovered the downregulation of *ahyR* with carvacrol treatment in two separate studies, suggesting the involvement of AI-1 QS inhibition (Wang et al. 2022; Lu et al. 2023).

**Table 3:** A table summarizing studies that have been performed on QS inhibition in *Aeromonas* to date

Inhibitor	Pathogen	Infection model	Outcome	Reference
Chestnut honey	*A*. *hydrophila*	NA	AHL degradation. Decreased biofilm	Truchado et al. (2009)
Tigonella foeum-graecum L. (Fenugreek)	*A*. *hydrophila*	*C*. *elegans*	Decreased biofilm, motility, and protease production. Increased survival in vivo	Husain et al. (2015)
Tannic acid	*A*. *hydrophila*	Fish	Downregulated *ahyRI* expression. Decreased hemolysis, motility, biofilm, and virulence in vivo	Patel et al (2017)
AHL lactonase AiiA_A196_	*A*. *veronii*	NA	Decreased motility and protease production	Gui et al (2017)
Curcumin liposomes	*A*. *sobria*	NA	Decreased siderophore production, motility, protease activity, and biofilm. In silico binding to AhyI	Ding et al. (2017)
Mangifera indica L. (mango leaf)	*A*. *hydrophila*	NA	Decreased biofilm formation	Husain et al. (2017)
(-)-Dimethyl 2,3-O-isopropylidene-l-tartrate	*A*. *hydrophila*	NA	Reduced AI-2 production. Reduced bacterial growth rate	Ali et al. (2018)
Curcumin liposomes	*A*. *hydrophila*	NA	Decreased biofilm, protease production, and motility	Ding et al. (2018)
Compounds on scaffold alkyl-quinoxalin-2(1H)-one	*A*. *caviae*	NA	Decreased biofilm	Blöcher et al. (2018)
AHL Lactonase AiiK	*A*. *hydrophila*	NA	AHL degradation. Decreased biofilm, motility, proteolytic, and hemolytic activity	Dong et al. (2020a, b)
*Bacillus licheniformis* T-1	*A*. *hydrophila*	Fish	Reduced pathogenicity and increased survival in vivo	Chen et al. (2020)
Curcumin	*A*. *hydrophila*	NA	Downregulated *ahyRI* expression. Decreased bacterial biofilm formation, motility, and protease activity	Mangoudehi et al. (2020)
Thymol	*A*. *hydrophila*	In vitro – A549 cells; in vivo – fish	Downregulated *aerA* and *ahyRI* expression. Decreased cell cytotoxicity. Increased survival in vivo	Dong et al. (2020a, b)
Methyl anthranilate	*A*. *sobria*	NA	Downregulated *ahyRI* expression. Decreased AHL production. Decreased biofilm, motility, and protease activity. In silico competitive binding with AhyR	Li et al. (2020)
Genistein	*A*. *hydrophila*	In vitro – A549 cells; in vivo – fish	Downregulated *ahyRI* expression. Decreased biofilm and aerolysin production. Increased cell viability. Increased survival in vivo	Dong et al. (2021)
Esculetin	*A*. *hydrophila*	NA	Downregulated *ahyRI* and *luxS* and upregulated *litR* expression. Decreased biofilm, motility, hemolysis, and protease production	Sun et al. (2021)
Sanguinarine	*A*. *hydrophila*	Fish	Decreased biofilm production, inhibition of aerolysin	Zhang et al. (2022b)
Carvacrol	*A*. *hydrophila*	Fish	Downregulated *ahyR* expression. Decreased biofilm, hemolysis, and protease activity	Wang et al (2022)
N-cis-octadec-9Z-enoyl-L-homoserine lactone	*A*. *hydrophila*	NA	Reduced AI-1 production	Ali et al (2022)
*Streptomyces sp*. *SH5*	*A*. *hydrophila*	Fish	Increased flora diversity, immune response, and survival in vivo. Downregulated *aerA*, *act*, *ast*, *hlyA*, *alt*, and *ahyRI* expression	Liang et al. (2022)
Klebsiella and Enterobacter commensals	*A*. *hydrophila*	Fish	Decreased AHL production. Increased survival in vivo	Omar et al. (2023)
Cinnamaldehyde	*A*. *hydrophila*	In vitro – A549; in vivo – Fish	Downregulated *ahyRI* expression. Decreased AHL production. Decreased biofilm, motility, hemolysis, and protease production. Reduced cytotoxicity in vitro. Increased survival in vivo	Li et al. (2023)
Hesperidin methyl chalcone (HMC)	*A*. *hydrophila*	Fish	Downregulated *ahyR* expression, decreased biofilm, motility, hemolysis, and protease production. Decreased bacterial loads in vivo	Roshni et al. (2023)
Resveratrol	*A*. *hydrophila*	Fish	Decreased biofilm and hemolysis. Downregulated QS-related gene expression	Qin et al. (2023b)
Carvacrol	*A*. *hydrophila*	NA	Decreased AHL production and downregulated ahyRI expression	Lu et al. (2023)

High-throughput screening for QS inhibitory molecules is becoming increasingly common to discover novel QS inhibitors (Zhang et al. 2022c). In silico methodologies can be used to do this via predicted 3-dimensional structures of the proteins involved. In that vein, the protein structure of AhyI was predicted and functionally characterized. Following that, the AI-1 synthase inhibitor N-cis-octadec-9Z-enoyl-L-homoserine lactone was then identified using high-throughput virtual screening. When tested, this molecule was found to effectively inhibit AI-1 activity at a concentration of 40 mM (Ali et al. 2022). Work has also been carried out to uncover novel AI-2 QS inhibiting compounds. In silico modeling of the AI-2 QS LuxS protein structure facilitated the prediction of putative binders and inhibitors of LuxS. From those predictions, a compound named ( −)-dimethyl 2,3-O-isopropylidene-l-tartrate was chosen for downstream testing, and it was shown to be an effective AI-2 QS inhibitor also at a concentration of 40 μM. Furthermore, *A*. *hydrophila* growth was significantly reduced when AI-2 QS inhibitor was added in conjunction with 1 mg/ml of oxytetracycline treatment (Ali et al. 2018). The use of in silico predictive models can more efficiently inform the discovery/design of novel drug candidates, especially when used synergistically with sub-lethal concentrations of bonafide antibiotics.

Alternatively, some research efforts have focused on exploring the role of commensal bacteria in pathogenic QS degradation. For example, one study reported that co-culturing *A*. *hydrophila* with three separate fish-gut-derived probiotic bacteria decreased AHL production by *A*. *hydrophila* and increased survival in an in vivo tilapia model when challenged (Omar et al. 2023). Similar results are found when a *Streptomyces* commensal (Liang et al. 2022) and a *Bacillus* commensal (Chen et al. 2020) were used in a zebrafish model and challenged with *A*. *hydrophila*. Because these studies have all focused on aquaculture and fish models, the efficacy of this technique in a mammalian model and the use of human commensals remain unexplored.

The emerging body of literature strongly suggests that blocking QS can be an effective way to reduce *Aeromonas-*related disease burden in aquaculture. Unfortunately, its potential in humans is left almost entirely unexplored. Given that all in vivo QS inhibition studies to date have been performed in a fish model of infection, a more clinically relevant understanding of many of these QS inhibitors needs to be established. Toward that end, some QS inhibitors have been tested in mammalian cell lines. Resveratrol, while effective in fish, demonstrated cytotoxicity in the murine macrophage cell line J774A.1 at higher concentrations (Qin et al. 2023b). One group observed that the plant extract, sanguinarine, was successful at reducing QS-regulated virulence factors like biofilm production and hemolysis at concentrations of 4 mg/ml. Sanguinarine was found to provide significant protection to human A549 cells from aerolysin-induced cell injury at this same concentration (Zhang et al. 2022b). In fact, thymol, genistein, and cinnamaldehyde have all demonstrated anti-QS activity and reduced cytotoxicity in human A549 cells (Dong et al. 2020a, b; Dong et al. 2021; Li et al. 2023). So, what little evidence we do have of these inhibitors in mammalian cell lines is, at best, varied. To further complicate the situation in a mammalian model, it has been shown that pre-treating mice with QS AI-1 signaling molecule AHL before challenging with *A*. *dhakensis* prevents clinical sequelae and produces increased survival in a septicemic model of infection (Khajanchi et al. 2011). While QS inhibition is a demonstrably effective way to reduce pathogenicity in a fish model, more research needs to be performed to ascertain its effectiveness in humans. Furthermore, the vast majority of QS inhibitory studies have been conducted using *A*. *hydrophila* as the model pathogen. Studies on this topic need to shift from the discovery of new compounds with anti-QS activity to fully characterizing the known compounds in different *Aeromonas* spp. and infection models.

## Conclusion

*Aeromonas* spp. are well-established aquatic fish and emerging human pathogens (Fernández-Bravo and Figueras 2020; Hayatgheib et al. 2020). Control of these aquatic pathogens is critical to both protecting aquaculture and its associated economy, as well as to prevent potential human disease. When considering treatment, antibiotic resistance is a major global threat in all bacterial pathogens, and *Aeromonas* infections are no exception. In fact, antibiotic resistance of various types has been globally documented in *Aeromonas* spp. (Bargui et al. 2023; Bhaskar et al. 2015; Hayes et al. 1994). Unfortunately, *Aeromonas* spp. have been shown to acquire resistance from other pathogens as well as readily share resistance with other species/strains (Canellas et al. 2023; Goñi-Urriza et al. 2000; Igbinosa et al. 2015). On account of this, *Aeromonas*, being aquatic by nature, has rendered aquatic environments including treated waters, significant reservoirs for AMR acquisition and retention (Drk et al. 2023; Rhodes et al. 2000). This is particularly challenging given the potential economic impact of disrupting the aquaculture industry. Furthermore, in serving as potential reservoirs for AMR, these hardy aquatic pathogens can not only cause drug-resistant human diseases but also facilitate the spread of AMR to other unrelated bacterial pathogens. Viewed in this light, AMR *Aeromonas* could become a major contributor to the problem, setting the stage for nightmarish scenarios associated with the post-antibiotic era.

When facing antimicrobial challenges, *Aeromonas* spp. employ numerous AMR strategies of evasion including mutations of the drug targets themselves and, in some cases, expression of specific antibiotic degrading enzymes (if their encoding genes are present) (Piccirilli et al. 2022; Rasmussen and Bush 1997; Sinha et al. 2004). *Aeromonas* spp. also employ a number of highly effective broad-spectrum AMR strategies such as efflux pumps and drug uptake inhibition (Wu et al. 2023; Zhang et al. 2022d). When attempting to acquire a clear picture of AMR pathways involved in antimicrobial evasion, some ambiguity is found on account of pathway crosstalk, synergy, and even, at times, antagonism. Indeed, proteomic analysis reveals the truly complicated nature of *Aeromonas*’ phenotypic resistance to antibiotic-associated stress which further complicates the issue (Li et al. 2021; Liu et al. 2023). Because there are so many different strategies employed to this effect, a complete understanding of antibiotic resistance in *Aeromonas* spp. cannot be reached by examining each strategy/pathway independently. As a result, this remains a fruitful area of study.

*Aeromonas spp*. have also been shown to harbor AI-1, AI-2, and AI-3 QS systems (Khajanchi et al. 2012; Kozlova et al. 2008; Swift et al. 1997). All three systems are essential for the regulation of each other and overall virulence potential (Talagrand-Reboul et al. 2017). Without them, infection cannot be established (Natrah et al. 2012). Because of its essential and ubiquitous nature, QS may be a point of vulnerability to be exploited from a therapeutic perspective. In that vein, many pharmaceutical/natural alternatives to conventional antibiotics/antimicrobials therapies have been proposed specifically targeting the QS systems in *Aeromonas* spp. to great effect (Li et al. 2023; Patel et al. 2017; Qin et al. 2023b; Roshni et al. 2023; Tan et al. 2019; Wang et al. 2022). The limitation of these therapeutic studies has been their primary focus on aquaculture and fish health. In large part, these studies have employed in vivo fish models with the ecological and economic health of fish culturing in mind, thereby limiting data available on efficacy for use in a human context. Unfortunately, *Aeromonas* spp. infections continue to pose a challenge to human health, and when considering multiple-drug-resistant *Aeromonas* spp. infections, treatment alternatives become critical. In fact, the post-antibiotic era has created a pressing need for the development of alternative therapeutics for bacterial infections extending well beyond those caused by *Aeromonas* spp. alone. Ultimately, additional investigations on the use of these alternative therapeutics in a clinically relevant context are warranted for *Aeromonas* spp. infections and beyond.

## Funding

This work was supported by the National Institutes of Health (NIH) R21 AI135453 grant and the pilot grants from the Institute for Human Infections and Immunity, UTMB, as well as the John S. Dunn chair in Global health Endowment to AKC, and the National Science Foundation (NSF) HRD-1345173 (JAR), HRD-1400962 (JAR), and HRD-1622993 (JAR) awards. The authors declare there are no conflicts of interest.

## Declarations

This study was funded by the US National Institutes of Health R21 AI135453, as well as by the John S. Dunn chair in Global health Endowment and the US National Science Foundation (HRD-1345173, HRD-1400962, and HRD-1622993).

## Human and animal studies

This article does not contain any studies with either animal or human subjects.
